# Negation recognition in clinical natural language processing using a combination of the NegEx algorithm and a convolutional neural network

**DOI:** 10.1186/s12911-023-02301-5

**Published:** 2023-10-13

**Authors:** Guillermo Argüello-González, José Aquino-Esperanza, Daniel Salvador, Rosa Bretón-Romero, Carlos Del Río-Bermudez, Jorge Tello, Sebastian Menke

**Affiliations:** 1 MedSavana SL, Madrid, 28004 Spain; 2 Savana Research, Madrid, SL 28004 Spain; 3 Faculty of Medicine and Health Sciences, University of Barcelona, Barcelona, 08007 Spain; 4 Statistics and Operations Research, University of Oviedo, Oviedo, 33003 Spain

**Keywords:** Negation, NegEx, CNN, Electronic health records, Clinical Natural Language Processing

## Abstract

**Background:**

Important clinical information of patients is present in unstructured free-text fields of Electronic Health Records (EHRs). While this information can be extracted using clinical Natural Language Processing (cNLP), the recognition of negation modifiers represents an important challenge. A wide range of cNLP applications have been developed to detect the negation of medical entities in clinical free-text, however, effective solutions for languages other than English are scarce. This study aimed at developing a solution for negation recognition in Spanish EHRs based on a combination of a customized rule-based NegEx layer and a convolutional neural network (CNN).

**Methods:**

Based on our previous experience in real world evidence (RWE) studies using information embedded in EHRs, negation recognition was simplified into a binary problem (‘affirmative’ vs. ‘non-affirmative’ class). For the NegEx layer, negation rules were obtained from a publicly available Spanish corpus and enriched with custom ones, whereby the CNN binary classifier was trained on EHRs annotated for clinical named entities (cNEs) and negation markers by medical doctors.

**Results:**

The proposed negation recognition pipeline obtained precision, recall, and F1-score of 0.93, 0.94, and 0.94 for the ‘affirmative’ class, and 0.86, 0.84, and 0.85 for the ‘non-affirmative’ class, respectively. To validate the generalization capabilities of our methodology, we applied the negation recognition pipeline on EHRs (6,710 cNEs) from a different data source distribution than the training corpus and obtained consistent performance metrics for the ‘affirmative’ and ‘non-affirmative’ class (0.95, 0.97, and 0.96; and 0.90, 0.83, and 0.86 for precision, recall, and F1-score, respectively). Lastly, we evaluated the pipeline against two publicly available Spanish negation corpora, the IULA and NUBes, obtaining state-of-the-art metrics (1.00, 0.99, and 0.99; and 1.00, 0.93, and 0.96 for precision, recall, and F1-score, respectively).

**Conclusion:**

Negation recognition is a source of low precision in the retrieval of cNEs from EHRs’ free-text. Combining a customized rule-based NegEx layer with a CNN binary classifier outperformed many other current approaches. RWE studies highly benefit from the correct recognition of negation as it reduces false positive detections of cNE which otherwise would undoubtedly reduce the credibility of cNLP systems.

**Supplementary Information:**

The online version contains supplementary material available at 10.1186/s12911-023-02301-5.

## Background

Traditionally, clinical evidence has been generated through randomized clinical trials or conventional research methods that involve an expensive and time-consuming manual data collection. Clinical natural language processing (cNLP) tools represent a time- and cost-effective solution for the generation of real-world evidence (RWE) using readily available real-world data (RWD) [1]. A paramount source of RWD is present in unstructured free-text of clinical notes registered by health professionals in patients’ electronic health records (EHRs) [2, 3]. The accurate and automated recognition of clinical named entities (cNE) and their attributes is essential to enable the use of this valuable information for research purposes in a big data setting.

One of the big challenges in cNLP is the recognition of negated cNE since negation is common in clinical narrative and crucial for any practical interpretation of clinical text [4, 5]. Negation and speculation are usually expressed using common triggers such as “no”, “no sign of”, or “absence of”. Nevertheless, many instances of cNEs are negated or speculated using much more complex linguistic structures. Thus, to avoid nefarious consequences in healthcare, several approaches have been developed to solve the negation problem across different languages, ranging from rule-based methods to neural networks [6].

Rule-based approaches have been implemented in English [7], Spanish [8], French [9], German [10], and Swedish [11], among others, achieving good performance in specific tasks [12]. However, they do not generalize properly to arbitrary clinical text because everything not explicitly coded with rules is not detected [4]. This lack of generalizability of rule-based systems drove the development of machine learning systems, such as conditional random fields (CRF) classifiers, that are commonly used for negation recognition in different languages [6, 13, 14]. The latest advances in the field are based on deep neural network architectures that identify the tokens under the scope of a negation using word embeddings [6, 13, 14]. The attention-based bidirectional Long Short-term Memory (LSTM) networks [15, 16], hidden layer feed-forward neural networks [15] or convolutional neural networks (CNNs) [17, 18] reached state-of-the-art metrics [19].

In Spanish, Bi-LSTMs (bidirectional LSTMs) have been applied to detect negation cues [20], negation triggers [21] and negation scope [19]. The generalization capabilities of deep neural networks are related to the training data in such a way that improvements of the model (other than optimizing model parameters) are only achievable via training with additional data. This is especially difficult in cNLP due to the highly complex lexical and syntactic content of EHRs [5]. In addition, gaining access to EHRs to use them as training data is often hindered by data protection laws, which adds an additional barrier to model development in cNLP. Thus, the main problem of current approaches to automatically detect negation in Spanish clinical texts consists in the lack of data to train and thoroughly test these models to guarantee their generalization capacities.

In the light of the above, we approached the negation recognition in EHRs’ free-text by combining the benefits of rule-based approaches for the detection of common negation triggers with the outstanding performance of neural networks to deal with linguistically complex negation structures. Specifically, we combined a customized rule-based NegEx layer [22] with a CNN binary classifier and tested performance on internal and external gold standards. Even though Transformers [23] have dominated the research landscape in NLP in recent years, our results highlight commonly overlooked benefits to convolutions such as model quality, speed, floating point operations per second (FLOPs), and scalability [18].

## Methods

The negation entity recognition approach described in this study consisted of three main phases, namely (1) Negation corpus creation, (2) Negation recognition pipeline development, and (3) Negation recognition pipeline evaluation.

### Negation corpus creation

To create a representative negation corpus, cNEs were selected from a wide range of document types from different hospital services. In order to achieve a high quality negation corpus, the annotation of the negation entities was performed following an internal annotation guideline (Supplementary Information 1). Briefly, the following classes were considered during the annotation: (i) Affirmative (i.e., the linguistic presence of the cNE is supported), (ii) Negative (i.e., the linguistic presence of the cNE is negated), (iii) Speculated (i.e., the linguistic presence of the cNE is uncertain), and (iv) Recommended (i.e., the linguistic presence of the cNE is recommended). Based on the experience of our medical experts, the CNN model was simplified into a binary problem with the classes ‘affirmative’ and ‘non-affirmative’ (combining negation, speculation, and recommendation).

### Negation recognition pipeline development

The framework for negation recognition is based on the combination of a customized rule-based NegEx layer [22] and a CNN binary classifier [24]. NegEx is one of the first and most widely used cNLP libraries for negation recognition using a rule-based approach. NegEx analyzes a window size of five tokens around the entity and considers three types of negations, namely ‘preceding negations’, ‘following negations’, and ‘pseudo-negations’ (i.e., they seem to be negations, but do not actually negate the medical entity). Finally, termination terms, including conjunctions such as “but” that indicate the ending of the scope of a previous negation term, are detected.

**Fig. 1:**
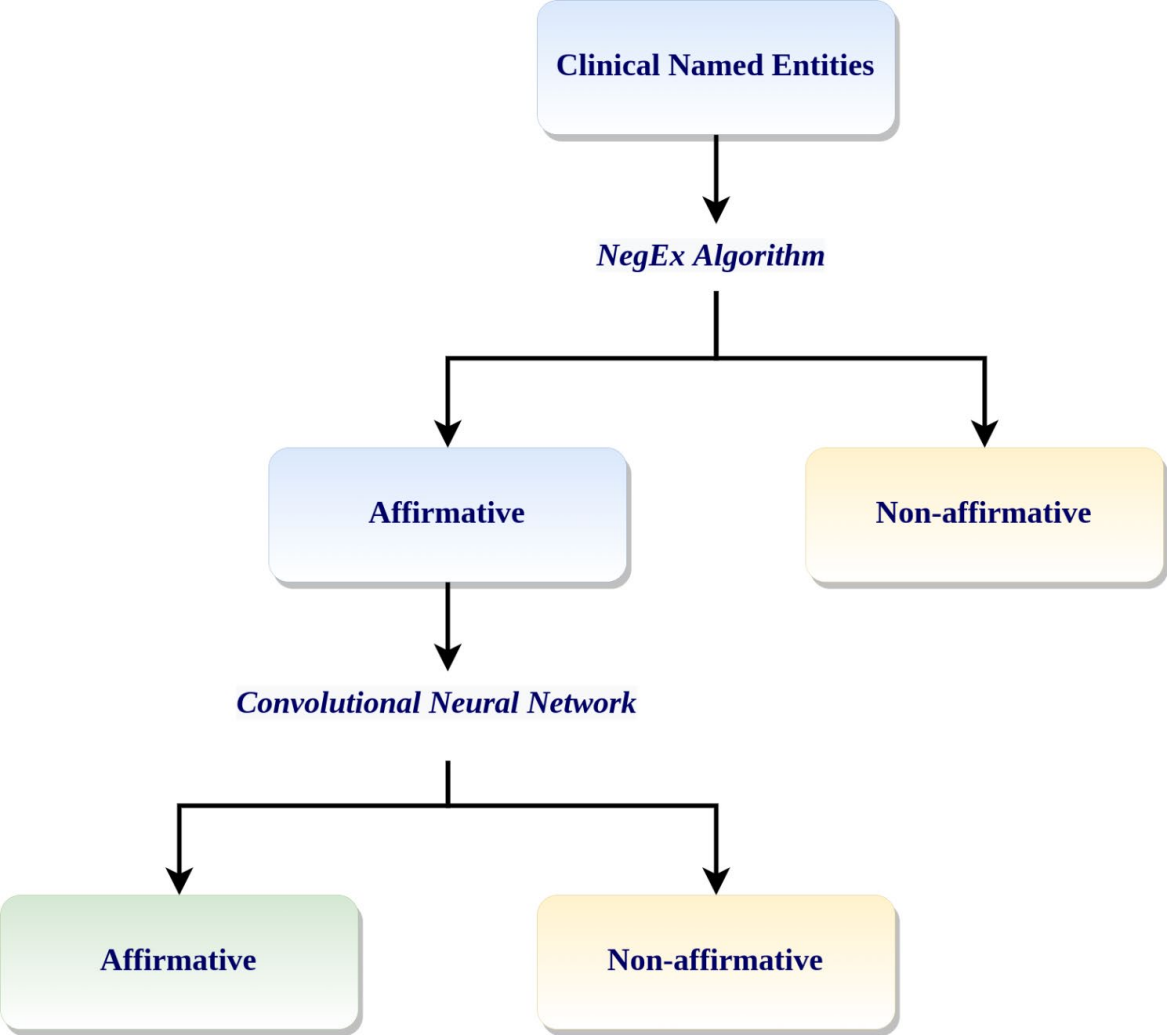
Negation recognition pipeline. A customized rule-based NegEx layer was placed before the CNN binary classifier. Any cNE classified as ‘affirmative’ serves as input to the CNN which makes the final decision about whether the cNE is really ‘affirmative’ or should be classified as ‘non-affirmative’

We adapted NegEx to the healthcare domain by refining and enriching a set of rules already designed [25]. This customized rule-based NegEx layer functions as the entry point of our pipeline and classifies cNE into ‘affirmative’ or ‘non-affirmative’. The cNEs that are classified as ‘affirmative’ serve as the input to the second layer of the pipeline, the CNN binary classifier. The schema of the pipeline is shown in Fig. 1.

We developed the CNN binary classifier using dense representations as features (embeddings) to solve negation recognition in cNEs. Most neural network language models are word-based and depend on a finite, predefined vocabulary. EHRs are written by medical doctors and other healthcare professionals that usually work under stressful conditions. This results in misspellings and heavy use of abbreviations which turn EHRs into very complex texts. This linguistic richness leads to a situation in which many words are not presented during model training, meaning they are out of vocabulary, which negatively affects the model performance. To address this problem, we trained a SentencePiece tokenizer in unigram mode [26] using the free-text of 30,000 EHRs (11,255,535 tokens) resulting in a subword vocabulary of 20,000 words.

**Fig. 2:**
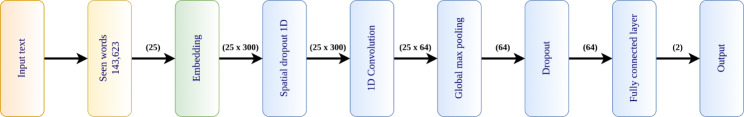
CNN model architecture. CNN binary classifier architecture for the prediction of cNEs into the ‘affirmative’ or ‘non-affirmative’ class

When the model is applied, each input text is tokenized and sentences are subsequenlty converted from a list of strings to a list of vocabulary indices of the tokenizer. This list of indices serves as input to the embedding layer of the model. The embedding layer converts the input into a dense real vector of fixed size and shape, one for each word in the tokenizer. These vectors are the input for the CNN composed by only one convolutional layer preceded by a spatial dropout layer and succeeded by a max pooling and dropout layers. Finally, a fully connected layer outputs the predicted label. The schema of this model is shown in Fig. 2.

### Negation recognition pipeline evaluation

**Fig. 3:**
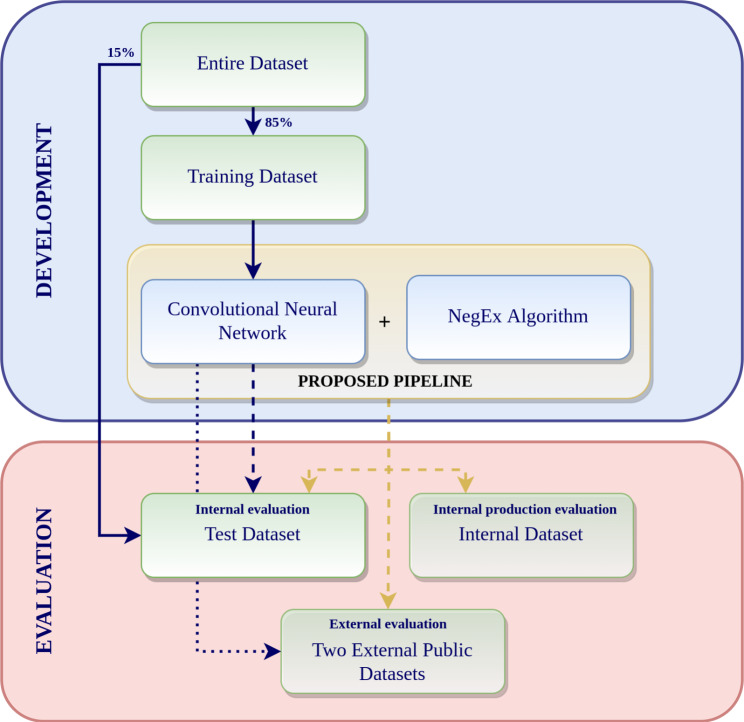
Development and evaluation schema. Workflow followed for the development and evaluation of the CNN binary classifier solely and in combination with the customized rule-based NegEx layer using internal as well as external datasets

The negation recognition pipeline evaluation was performed using our internally annotated negation corpus and two publicly available gold standards for negation in (a) a development environment and (b) a production environment with Apache Spark running on Amazon Web Services (AWS) infrastructure using Elastic Map Reduce (EMR) clusters (Fig. 3). Evaluation was performed for the CNN binary classifier solely and for the combination with the customized rule-based NegEx layer. Performance was measured using precision, recall, and F1-score metrics.

#### Internal evaluation

The CNN binary classifier was trained using our in-house annotated negation corpus. According to standard practice in machine learning model development, we applied a train-validation-test split of 85/7.5/7.5. The hyper parameters of the CNN were fine-tuned by training the model with a set of different values for learning rate, dropout, epochs, and batch size. Finally, the model parameters resulting in the best performance were selected as the final parametrization of the network. To assess the scalability of the proposed framework and validate its performance when dealing with large amounts of EHRs, we integrated it in Apache Spark, an open-source computing framework designed for large-scale distributed data processing. It provides advanced APIs in Java, Scala, Python, and R. It also supports some advanced components, including Spark SQL for structured data processing, MLlib for machine learning, GraphX for computing graphs, and Spark Streaming for real-time data processing [27]. Specifically, the Python API PySpark was used as we did all the development in Python. Apache Spark installs all dependencies across all the executors to allow parallelized inference and the iterator of batch function [28] enables processing of the input data in batches of records in each executor. For pipeline implementation, the *negspacy* library [29] was used for the rule-based part based of the NegEx algorithm and *MLFlow* [30] to load the previously trained CNN binary classifier. To launch the pipeline, we used Elastic MapReduce (EMR) cluster composed of 1 Master node (instance type m5.xlarge), 4 core nodes (r5.4xlarge) and 8 task nodes (r5.4xlarge). EMR is an AWS service that allows full control over preconfigured machine specifications and data being processed [31].

To assess the generalization capabilities of the negation recognition pipeline, we executed the whole pipeline against EHRs from a different data source distribution than the training corpus using Apache Spark. We mixed randomly selected EHRs with those that were selected based on specific cNEs that are frequently negated such as ‘diabetes’, ‘arterial hypertension’ or ‘hematuria’. A subset was manually annotated by medical doctors and the pipeline results were evaluated against those.

#### External evaluation

For the external evaluation, two public datasets were used: IULA, “IULA Spanish Clinical Record Corpus” [32] and the NUBes (“Negation and Uncertainty annotations in Biomedical texts in Spanish”) corpus [33]. The IULA corpus also contains negated phrases that are not from the medical domain which we filtered out, resulting in a final gold standard corpus of 1,172 cNE. We tested the CNN binary classifier solely and in combination with the customized rule-based NegEx layer on these negated cNE. The NUBes dataset contains negated as well as speculated cNE, which we merged together into the ‘non-affirmative’ class for comparison with our approach. To be consistent with our guidelines, we further excluded some entities that NUBes’ authors consider negated, but we did not. For instance, some lexical negations such as negative *(“negativo/a”)* and the morphological negations such as afebrile or asymptomatic *(“afebril”, “asintomático”).* Again we tested both the CNN binary classifier solely and in combination with the customized rule-based NegEx layer on the resulting reference corpus.

## Results

To ensure the generalization of the proposed negation recognition pipeline to any EHR written in Spanish, we carried out three evaluations, one using an internal dataset and two using public ones (Fig. 3). We tested the CNN binary classifier solely and in combination with the customized rule-based NegEx layer on the test split of our in-house annotated negation corpus. The combination of NegEx and CNN evaluated in a total of 203 testing examples yielded improved metrics (F1-score: 0.85) compared to only the CNN binary classifiers (F1-score: 0.83) (Table 1) for the ‘non-affirmative’ class.

**Table 1:** Internal evaluation metrics during development using only the Convolutional Neural Network (CNN) or the combination of the NegEx layer with the CNN (NegEx + CNN) applied on the test set containing 705 cNEs

		Precision		Recall		F1-score	
	N	CNN	NegEx + CNN	CNN	NegEx + CNN	CNN	NegEx + CNN
**Non-affirmative**	203	0.85	0.86	0.81	0.84	0.83	0.85
**Affirmative**	502	0.93	0.93	0.94	0.94	0.93	0.94
**Accuracy**	705					0.91	0.91
**Macro average**	705	0.89	0.90	0.88	0.89	0.88	0.89
**Weighted average**	705	0.91	0.91	0.91	0.91	0.91	0.91

To avoid any bias in the previous dataset and assess the generalization capabilities of our pipeline once the model was trained, we executed the whole pipeline with Apache Spark against productive data unseen by the model (3,481,673 EHRs containing 37,453,469 cNEs). The execution took 3 h and 15 min, corresponding to over 1 million EHRs processed per hour. Out of those, medical doctors manually annotated 6,710 cNE as ‘affirmative’ (n = 5,280) or ‘non-affirmative’ (n = 1,430). The evaluation of our approach against this annotated dataset resulted in an F1-score of 0.86 for the ‘affirmative’ class and 0.96 for the ‘non-affirmative’ class (Table 2).

**Table 2:** Evaluation metrics in an unseen and independently gathered dataset. To calculate internal evaluation metrics during development, we applied the Convolutional Neural Network (CNN) binary classifier solely or in combination with the customized rules-based NegEx layer (NegEx + CNN) to an unseen and independently gathered dataset composed of 6,710 manually annotated cNE (5,280 ‘affirmative’ vs 1,430 ‘non-affirmative’)

	N	Precision	Recall	F1-score
**Non-affirmative**	1430	0.90	0.83	0.86
**Affirmative**	5280	0.95	0.97	0.96
**Accuracy**	6710			0.91
**Macro average**	6710	0.92	0.90	0.91
**Weighted average**	6710	0.94	0.94	0.94

Next, we externally evaluated both the CNN binary classifier and the complete pipeline, which combined the customized rule-based NegEx layer and the CNN binary classifier, against two public clinical corpora for Spanish negation recognition: IULA corpus and NUBes corpus. The CNN binary classifier evaluated against IULA obtained a precision of 1.00, recall of 0.92, and F1-score of 0.96, which translates into a total of 98 misclassified entities out of 1,172. The combination of the customized rule-based NegEx layer with the CNN binary classifier resulted in a precision of 1.00, recall of 0.99, and F1-score of 0.99, with only 12 misclassified entities (Table 3).

**Table 3:** External evaluation metrics of the ‘non-affirmative’ class in IULA and NUBes corpus. External validation using the IULA Spanish Clinical Record Corpus with 1,172 and the NUBes corpus with 11,400 negated entities. For both, metrics are shown using the CNN model solely and in combination with the customized rule-based NegEx layer. As the IULA corpus only contains negated entities, only the ‘non-affirmative’ class is shown. The NUBes corpus only contains negated and speculated entities and both were considered ‘non-affirmative’. Only the ‘non-affirmative’ class is shown

		Precision		Recall		F1-score	
	N	CNN	NegEx + CNN	CNN	NegEx + CNN	CNN	NegEx + CNN
**IULA corpus**	1,172	1.00	1.00	0.92	0.99	0.96	0.99
**NUBes corpus**	11,400	1.00	1.00	0.76	0.93	0.86	0.96

Lastly, CNN binary classifier performance was evaluated against 11,440 negated entities in NUBes. Here, all entities that were annotated as negated or speculated were considered ‘non-affirmative’. Finally, we obtained a precision of 1.00, recall of 0.76, and F1-score of 0.86, being 2,765 entities misclassified. The combination of the customized rule-based NegEx layer with the CNN binary classifier resulted in a precision of 1.00, and improved recall and F1-score (0.93 and 0.96, respectively). Only 803 entities were misclassified in the process (Table 3).

## Discussion

To improve the negation recognition performance in RWE studies, we have built a novel cNLP pipeline combining a customized rule-based NegEx layer with a CNN binary classifier. This approach yielded state-of-the-art metrics in both internal and external evaluations thereby proving the usefulness of this combination for the negation recognition task in free-text of EHRs.

Negations are frequent in clinical texts making negation recognition a crucial element of any cNLP pipeline to avoid false positives in RWE studies. In addition to the high lexical variability present in EHRs’ free-text (i.e., frequent use of alternative medical forms, non-standard acronyms, variants in misspellings and punctuation errors) [19, 34], negation recognition is a complex task itself due to the multiple forms in which a negated term can appear [35, 36].

NegEx, one of the most popular rule-based algorithms for negation, has been widely used and adapted to languages other than English [10, 11, 22, 37]. In addition, deep learning approaches have been implemented to further improve negation recognition [17, 38, 39]. For instance, context-independent and context-dependent pretrained transformers models achieved an F-score performance of over 85% for negation recognition in medical text outperforming rule-based methods [40]. The authors analyzed the most frequent false negatives and false positives for negation and speculation recognition and concluded that the ambiguity of some grammatical structures led their model to misclassify some tokens resulting in a decreased performance [40].

To overcome this decrease in performance seen by others, we focused on avoiding the prediction of a cNE to be ‘affirmative’ when it is actually ‘non-affirmative’. When we added the customized rule-based NegEx layer before the CNN binary classifier, we observed an improvement of the negation recognition of the CNN binary classifier itself with a decrease of the CNN binary classifier’s error rate, and an increase of the specificity of the pipeline.

We reached an F1-score of 0.86 for the ‘non-affirmative’ class when applied on our internal test negation corpus. When applied to the two external databases IULA and NUBes, we obtained an F1-score of 0.99 and 0.96, respectively. The latter proves that our proposed approach outperformed current state-of-the-art methods in the healthcare field [41]. Interestingly, performance metrics of our negation recognition pipeline were better for the external datasets than the internal one, probably due to the linguistic complexity of our internal test negation corpus which covered a greater variability of negation expressions compared to the publicly available datasets.

The true potential of RWE studies lies in the use of large amounts of data to generalize research findings that could have an impact in clinical practice [1]. Therefore, cNLP models developed to extract information from EHRs in RWE need to fit into big data processing frameworks to achieve predictions in a reasonable amount of time. Here, we have shown that the integration of our negation recognition pipeline in Apache Spark manages to classify tens of millions of cNEs in a million of EHRs per hour. To the best of our knowledge, this is the first time a study presents results of a production pipeline using Apache Spark to address the inference of the negation recognition in cNEs at scale.

The proposed negation recognition pipeline presents some limitations. First, it only predicts the two classes ‘affirmative’ and ‘non-affirmative’, with the latter including speculated cNE. We preferred two balanced classes over having more classes to avoid noise caused by the ambiguity of grammatical structures which would finally lead to misclassified cNE. Future work could focus on detecting negation and speculation separately without compromising the overall performance. Second, our results are based on the combination of a customized rule-based NegEx layer with a CNN binary classifier and future work is needed to explore how other model architectures affect the performance in prediction and execution at scale. In this study, the CNN architecture has proven to be a good choice for the second layer of our negation recognition pipeline both in quality of predictions as well as performance in a production environment.

## Conclusion

We demonstrated that the combination of a customized rule-based NegEx layer with a CNN binary classifier results in a powerful, easy to adapt pipeline reaching state-of-the-art performance in negation recognition in cNLP. The application of such a negation recognition pipeline in RWE studies highly increases the confidence in the results obtained from downstream analyses that inform decision makers in the clinical domain. Furthermore, this architecture seamlessly integrates with a production pipeline for predictions at scale as is required in big data RWE studies.

### Electronic supplementary material

Below is the link to the electronic supplementary material.

Additional File 1: Annotation guidelines.

## Data Availability

Consent for publication of raw data not obtained and dataset could in theory pose a threat to confidentiality.

## Abbreviations

AWS: Amazon Web Services
bi-LSTM: Bidirectional long short-term memory
cNE: Clinical named entity
cNLP: Clinical Natural Language Processing
CNN: Convolutional neural network
EHR: Electronic health record
EMR: Elastic Map Reduce
FLOPs: Floating point operations per second
iula: IULA Spanish Clinical Record Corpus
KOL: Key Opinion Leaders
LSTM: Long short-term memory
NLP: Natural Language Processing
NUBes: Negation and Uncertainty annotations in Biomedical texts in Spanish
RWD: Real World Data
RWE: Real World Evidence
