# Supplementary material for: Negation recognition in clinical natural language processing using a combination of the NegEx algorithm and a convolutional neural network
Source: BMC Med Inform Decis Mak. 2023 Oct 13;23:216. doi: 10.1186/s12911-023-02301-5 (PMC10576331; doi:10.1186/s12911-023-02301-5)
Supplement: Supplementary file 1 — Additional File 1: Annotation guidelines. [file 12911_2023_2301_MOESM1_ESM.pdf]

DISCLAIMER: Translations from Spanish to English were only added to this supplement to enhance understanding of readers of the publication and were not present in the original annotation guidelines as annotations were performed on Spanish texts.

Negation-Speculation Annotation Guidelines

Subject

The subject of these guidelines is to describe the annotation task of the Negation-Speculation status of a **c**linical **N**amed **E**ntity (cNE) in a textual context.

A given cNE in a sentence or phrase can labeled as:

affirmative, i.e. the linguistic presence of the cNE in question is supported by the text;
negative, i.e. the linguistic presence of the cNE in question is negated;
speculated, i.e. the linguistic presence of the cNE in question is uncertain.
recommended, i.e. the linguistic presence of the cNE in question is recommended

It is very important to underline that the Negation-Speculation status of a cNE is understood from a **linguistic** perspective, **NOT** from a medical or any other interpretive perspectives. This means that medical relevance, justice or interpretation is **NOT** subject to the annotation and shall not be taken into account.

The reason for this linguistic approach is technical and is motivated by the manner in which statistical models are learning from text. An artificial neural network, or any other statistical modeling approach, applied to text disposes only of information which it can deduce from textual structures, such as occurrences and co-occurrences of words. The model does not dispose of any world-knowledge which could be used to interpret the text for its medical relevance. This means that whether a cNE refers to a patient or a family member, is not relevant for the detection of the speculation-negation status, because the semantic information is the same. In addition, also the temporal information is irrelevant, i.e. it is irrelevant whether a cNE is negated, affirmed or speculated in the present or in the past.

Note: We initially defined these classes to facilitate the annotation task for human annotators. However, due to the limited number of annotations in the "speculated" and "recommended" classes, we have decided to simplify the classification and consider only two classes: "affirmative" and "non-affirmative." The "non-affirmative" class encompasses the previously separate classes of "negative," "speculated," and "recommended".

Categories definition with examples

The annotators dispose of the following categories for the classification:

Affirmative

In this category we group the cNEs that are clearly affirmative and the cNEs that change their status during the sentence from negative to affirmative.

Yes

Basic linguistic affirmative category. Something exists, is or was present, has been detected, diagnosticated, etc. Irrelevant whether in the present or in the past.

Examples:
Context: “Paciente diabetico, hipertenso” (English translation: “Diabetic patient, hypertensive”)
cNE: diabetico (English translation: “diabetic”)
Negation-Speculation status: Yes

Context: “Paciente con antecedentes de cáncer de mama” (English translation: “Patient with a history of breast cancer”)
cNE: cancer de mama, (English translation: “breast cancer”)
Negation-Speculation status: Yes

Context: “Paciente alergico a la penicilina” (English translation: “Patient allergic to penicillin”)
cNE: alergico (English translation: “allergic”)
Negation-Speculation status: Yes

Context: "El paciente presenta cáncer de colon" (English translation: “The patient has colon cancer”)
cNE: "cáncer" (English translation: “cancer”)
Negation-Speculation status: Yes

No to Yes

When the status of a cNE changes from negated to affirmative in the same sentence. For instance, when something not present in the past is present now.

Examples:
Context: “Paciente sin síntomas de isquemia que inicia hoy con dolor retroesternal.” (English translation: “Patient without symptoms of ischemia who started today with retrosternal pain”)

cNE: “isquemia” (English translation: “ischemia”)
Negation-Speculation status: No to Yes

Context: “Paciente crónico que se encontraba libre de analgesicos que inicia hoy con tramadol” (English translation: “Chronic patient who was free of analgesics who starts today with tramadol”)
cNE: "analgesicos" (English translation: “analgesics”)
Negation-Speculation status: No to Yes

Negative

In this category we group the cNEs that are clearly non-affirmative and the cNEs that change their status during the sentence from affirmative to negative.

No

The cNE in question is negated, determined as not present, not existing or not applicable.

Examples:

Context: “Paciente que niega fiebre u otro síntoma asociado.” (English translation:”Patient who denies fever or other associated symptoms.”)
cNE: “fiebre” (English translation: “fever”)
Negation-Speculation status: No

Context: “Se descarta derrame pericárdico” (English translation: “Pericardial effusion ruled out”)
cNE: “derrame pericardico” (English translation: “pericardial effusion”)
Negation-Speculation status: No

Context: “No se evidencian lesiones óseas"" (English translation: “No bone lesions are evident”)
cNE: “lesiones oseas” (English translation: “bone lesions”)
Negation-Speculation status: No

Yes to No

When the status of a cNE changes from affirmative to negated. For instance, when something present in the past is not present now.

Examples:

Context: “Paciente en seguimiento por reagudización de EPOC que al momento de la consulta se encuentra asintomático” (English translation: “Patient under follow-up for COPD exacerbation who at the time of consultation is asymptomatic”)
cNE: “reagudización de EPOC” (English translation: “COPD exacerbation”)
Negation-Speculation status: Yes to No

Speculated

In this class we group all types of speculations about a CNE, basically two: conditional and uncertain.

Speculation - conditional

Relates the presence or absence of a certain cNE to a condition, or where the cNE in question is the condition for some other appearance.

Examples:

Context: "En caso de presentar dolor, concurrir a nueva evaluación." (English translation: “In case of pain, attend a new evaluation”)
cNE: "dolor" (English translation: “pain”)
Negation-Speculation status: Speculation - conditional

Context: "Si la tos persiste, incrementar la dosis de ventolin a 2 inhalaciones cada 8hs." (English translation: “If the cough persists, increase the dose of ventolin to 2 inhalations every 8 hours.”)
cNE: "ventolin" (English translation: “ventolin”)
Negation-Speculation status: Speculation - conditional
Comment: here the cNE in question is conditioned by some other occurrence.

Context: "Si la tos persiste, incrementar la dosis de ventolin a 2 inhalaciones cada 8hs." (English translation: “If the cough persists, increase the dose of ventolin to 2 inhalations every 8 hours.”)
cNE: "tos" (English translation: “cough”)
Negation-Speculation status: Speculation - conditional
Comment: here the cNE in question conditions another occurrence.

Context: "De persistir con mareos, se consultará con neurologia." (English translation: “If dizziness persists, neurology will be consulted”)
cNE: "mareos" (English translation: “dizziness”)
Negation-Speculation status: Speculation - conditional

Speculation - Uncertainty

The condition of a cNE is uncertain or not yet definite

Examples:

Context: "Se solicita PCR para confirmar Covid-19." (English translation: “PCR is requested to confirm Covid-19”)
cNE: "Covid-19" (English translation: “Covid-19”)
Negation-Speculation status: Speculation - Uncertainty

Context: "Paciente en protocolo de estudio para mieloma múltiple." (English translation: “Patient in study protocol for multiple myeloma”)
cNE: "mieloma multiple" (English translation: “multiple myeloma”)
Negation-Speculation status: Speculation - Uncertainty

Context: "Paciente con sospecha de hepatocarcinoma" (English translation: “Patient with suspected hepatocarcinoma”)
cNE: "hepatocarcinoma" (English translation: “hepatocarcinoma”)
Negation-Speculation status: Speculation - Uncertainty

Recommended

A future event or a recommendation for the future.

Examples:
Context: "Se recomienda dejar de fumar." (English translation: “It is recommended to quit smoking.”)
cNE: "dejar de fumar" (English translation: “quit smoking”)
Negation-Speculation status: Recommendation

Context: "Se recomienda la realización de actividad física" (English translation: “Physical activity is recommended”)
cNE: "actividad física" (English translation: “physical activity”)
Negation-Speculation status: Recommendation

Context: "Se prescribe paracetamol cada 8 horas" (English translation: “Paracetamol is prescribed every 8 hours.”)
cNE: paracetamol (English translation: “paracetamol”)
Negation-Speculation status: Yes

Be aware!

Remember, it's a linguistic model. Thus, some medical terms and their meaning could be confusing when categorize to one of the classes, for instance:

Context: “Paciente afebril” (English translation: “Patient not feverish”)
cNE: afebril (English translation: “not feverish”)
Negation-Speculation status: Yes

Even though the medical condition “fever” is negated through the word “afebril”, the phrase itself is affirmative, the patient is afebrile.

Context: “Paciente asintomatico” (English translation: “Asymptomatic patient”)
cNE: asintomatico (English translation: “asymptomatic”)
Negation-Speculation status: Yes

Again, even though the patient has no symptoms, the phrase itself is affirmative. The patients is asymptomatic
